# Supplementary material for: The interactome of intact mitochondria by cross-linking mass spectrometry provides evidence for coexisting respiratory supercomplexes
Source: Mol Cell Proteomics. 2017 Dec 8;17(2):216–32. doi: 10.1074/mcp.RA117.000470 (PMC5795388; doi:10.1074/mcp.RA117.000470)
Supplement: Supplemental Data [file supp_RA117.000470_133922_0_supp_23978_fzffwf.zip › spectra_annotation/mito_spectra_annotation/61-1-2-1-2-1.pdf]

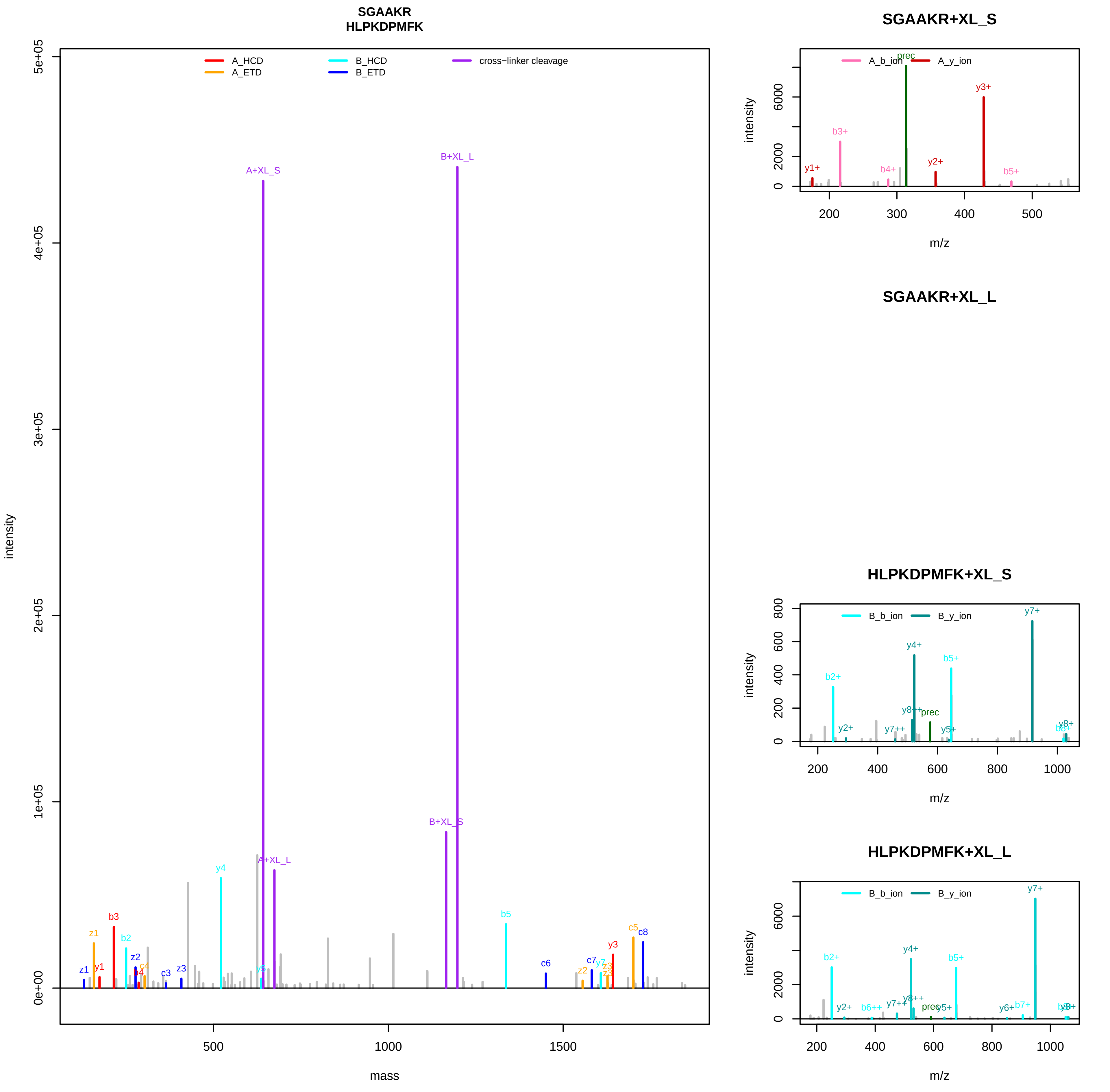

SGAAKR
HLPKDPMFK
A_HCD
A_ETD
B_HCD
B_ETD
cross−linker cleavage
A+XL_S
B+XL_L
B+XL_S
A+XL_L
intensity
mass
0e+00
1e+05
2e+05
3e+05
4e+05
5e+05
500
1000
1500
z1 y1 z1 b3 b2 z2 b4 c4 c3 z3 y4 y5 b5 c6 z2 c7 y7 z3 z3 y3 c5 c8
SGAAKR+XL_S
A_b_ion
A_y_ion
prec
y1+ b3+ b4+ y2+ y3+ b5+
SGAAKR+XL_L
HLPKDPMFK+XL_S
B_b_ion
B_y_ion
b2+ y2+ y7++ y8++ y4+ prec b5+ y5+ y7+ y8+ b8+
HLPKDPMFK+XL_L
b2+ y2+ b6++ y7++ y8++ y4+ prec y5+ b5+ y6+ b7+ y7+ y8+
m/z
